# The role of pentraxin3 in plasma and bronchoalveolar lavage fluid in COPD patients with invasive pulmonary aspergillosis

**DOI:** 10.1186/s12890-021-01793-z

**Published:** 2021-12-16

**Authors:** Qian He, Ming Zhang, Chunlai Feng

**Affiliations:** Department of Respiratory and Critical Care Medicine, Third Affiliated Hospital of Soochow University, Changzhou, 23000 China

**Keywords:** Pentraxin 3, Invasive pulmonary aspergillosis, Bronchoalveolar lavage fluid, Galactomannan

## Abstract

**Background:**

The use of galactomannan (GM) testing in plasma and bronchoalveolar lavage fluid (BALF) has improved the diagnosis of invasive pulmonary aspergillosis (IPA) in patients with chronic obstructive pulmonary disease (COPD); however, the high false-positive rate leads to overdiagnosis. Pentraxin 3 (PTX3) as an indicator of inflammation plays an important role in resistance to *Aspergillus* infections. This study aimed to investigate the diagnostic value of PTX3 for diagnosing IPA with COPD.

**Methods:**

We retrospectively collected data on patients with suspected COPD and IPA who had been hospitalized in the Third Affiliated Hospital of Soochow University between September 2017 and November 2020. PTX3 and GM were measured using enzyme-linked immunosorbent assays.

**Results:**

A total of 165 patients were included in the study, of whom 35 had confirmed or probable IPA. The remaining 130 patients served as controls. The median plasma and BALF PTX3 levels were significantly higher in patients with IPA than in control patients (3.74 ng/mL vs. 1.29 ng/mL, *P* < 0.001; and 3.88 ng/mL vs. 1.58 ng/mL, *P* < 0.001 in plasma and BALF, respectively). The plasma GM, plasma PTX3, BALF GM, and BALF PTX3 assays had sensitivities of 60.0%, 77.1%, 78.6%, and 89.3%, respectively, and specificities of 73.8%, 69.2%, 80.7%, and 77.1%, respectively. The sensitivity of PTX3 in plasma and BALF was higher than that of GM. However, the specificity of PTX3 and GM did not differ significantly between the IPA group and the control group. The specificity of the assays for the diagnosis of IPA was > 90% in patients who were PTX3-positive and GM-positive in plasma and BALF.

**Conclusions:**

BALF and plasma PTX3 levels were significantly higher in COPD patients with IPA. The sensitivity of PTX3 was superior to that of GM for diagnosing IPA in patients with COPD. The combination of GM and PTX3 is useful for the diagnosis of IPA in patients with COPD.

## Background

Invasive pulmonary aspergillosis (IPA) is a serious opportunistic infection caused by *Aspergillus* spp. In recent years, the incidence of IPA has gradually increased in patients with chronic obstructive pulmonary disease (COPD). Current research suggests that the occurrence of pulmonary *Aspergillus* infection in patients with COPD is related to the long-term use of glucocorticoids and antibiotics. Patients with COPD and pulmonary invasive aspergillosis often present with atypical symptoms and imaging [1]. Therefore, the early diagnosis of these patients is difficult, and the fatality rate is high [2]. The Infectious Diseases Society of America guidelines (IDSA) recommend testing plasma and bronchoalveolar fluid lavage (BALF) for galactomannan (GM) for the diagnosis of IPA [3]. However, some studies have found that the diagnostic value of the GM test in patients with non-neutropenic IPA, including among patients with COPD, is limited [4]. Therefore, it is necessary to identify adjunct biomarkers for the diagnosis of IPA.

Pentraxin 3 (PTX3), a member of the family of long pentraxins, is produced by dendritic cells, epithelial cells, endothelial cells, and macrophages at the sites of inflammation [5]. A study has shown that PTX3 knockout mice are more susceptible to *Aspergillus* infection, because of reduced PTX3 production [6]. A recent report described a link between PTX3 polymorphisms and susceptibility to aspergillosis in patients undergoing hematopoietic stem-cell transplantation and lung transplant recipient [7, 8]. Our previous research found that COPD patients with pulmonary aspergillosis have a high prevalence of PTX3 polymorphisms. In patients with COPD, plasma PTX3 levels are significantly increased in patients with IPA [9]. However, we did not evaluate the diagnostic value of PTX3 for IPA in our previous study. In patients with lung infections, BALF is a more direct indicator of lung inflammation than blood. This provides a new potential biomarker for the clinical diagnosis of IPA. Thus, in this study, we evaluated the diagnostic value of BALF and plasma PTX3 levels in COPD patients with IPA.

## Methods

### Patients

The medical records of patients with COPD admitted to Third Affiliated Hospital of Soochow University (Changzhou First People’s Hospital) from September 2017 to November 2020 with suspected IPA, were analyzed retrospectively. Steroid treatment was defined as oral or inhaled steroid therapy for patients, administered for at least 3 months.

The inclusion criteria were as follows: (1) A diagnosis of COPD (Based on dyspnea, chronic cough or sputum production and/or a history of exposure to risk factors for the disease, and post-bronchodilator FEV1/FVC less than 0.70 confirms the presence of persistent airflow limitation). Pulmonary function and disease severity were determined according to the Global Initiative for Chronic Obstructive Lung Disease Guidelines (GOLD, updated 2017) [10]. Subjects with a forced expiratory volume in 1 s of ≥ 80%, between 50–79%, 30–49%, and < 30% of the predicted values were designated as GOLD 1, 2, 3, and 4, respectively. (2) Symptoms of a respiratory infection such as fever, cough, sputum, dyspnea, or hemoptysis which had not been relieved after treatment with broad-spectrum antibiotics. (3) A chest computed tomography (CT) scan showing lung consolidation, with or without a halo sign, pulmonary nodules, or cavitary lesions.

The exclusion criteria were as follows: (1) a previous hematopoietic stem-cell or solid-organ transplant or neutropenia; (2) a diagnosis of asthma, bronchiectasis, post-tuberculosis sequelae, lung cancer, or other diffuse lung parenchymal disease.

### Collection of bronchoalveolar and plasma samples and measurement of galactomannan and pentraxin 3

Peripheral blood was collected from each subject before treatment initiation. Bronchoscopy was performed by a bronchoscopist with more than 3 years of experience in bronchoscopy. CT was used to locate the segment or subsegmental bronchus of the lesion. This area was rinsed twice with 50 mL saline. BALF was collected in a sterile tube and sent to the laboratory.

Plasma and BALF PTX3 levels were measured using an enzyme-linked immunosorbent assay kit according to the manufacturer’s protocol (DPTX30, Quantikine Human Pentraxin 3 Immunoassay, R&D, Abingdon, UK). The plasma and BALF samples were also tested for *Aspergillus* GM antigen using a double-sandwich ELISA (Platelia Aspergillus Kit, Bio-Rad Laboratories, CA, USA), performed according to the manufacturer’s instructions.

### Statistical analysis

Continuous variables were expressed as the mean ± standard deviation or the medians and interquartile ranges (IQR), according to their distribution. Categorical variables were expressed as proportions. For count data and categorical variables, the chi-squared test or Fisher’s exact test was used to compare groups, and for continuous variables, Student’s t-test or the Mann–Whitney U-test were used to compare groups, depending on whether or not the data were normally distributed. All data were analyzed using SPSS version 19.0 (IBM Corp, Armonk, NY, USA). A *P* value < 0.05 was taken to indicate statistical significance. Receiver-operating characteristic (ROC) curve analysis was used to determine the optimal cutoff value for GM and PTX3 for diagnosing IPA.

## Results

### Patient characteristics

**Fig. 1:**
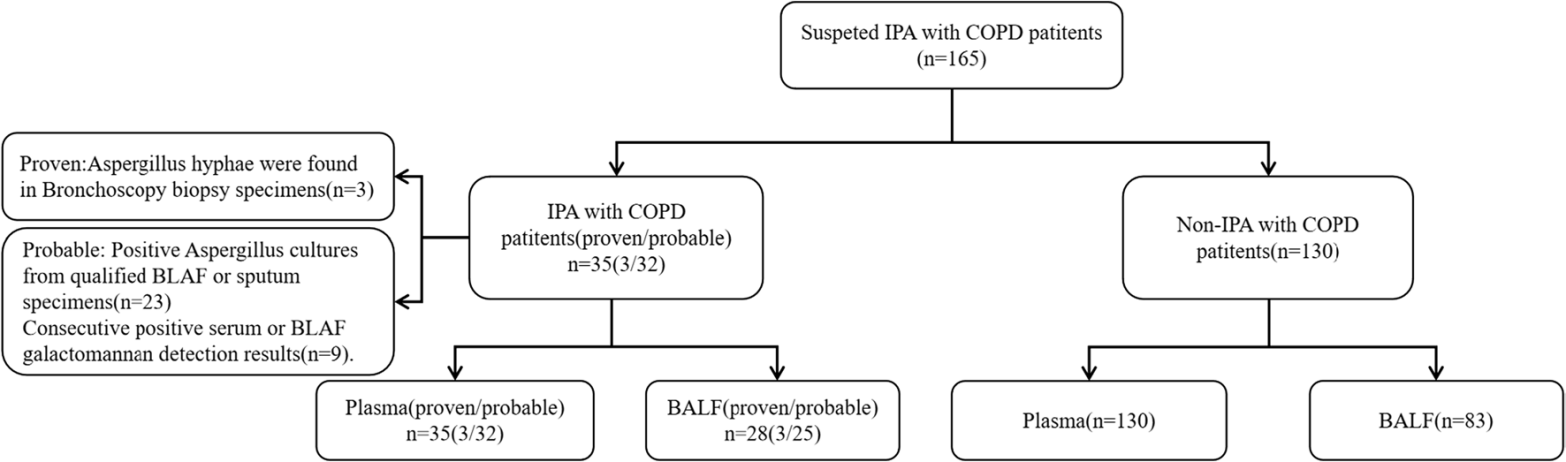
Study flow diagram

A total of 165 patients were included, of whom 35 were diagnosed with IPA and COPD according to the Infectious Diseases Society of America guideline criteria (3 confirmed; 32 probable), and 130 COPD patients without IPA served as controls (Fig. 1). No significant differences were observed in terms of sex, age, smoking history, and pulmonary function between the case and control groups (Table 1). A significantly greater proportion of patients in the IPA group used corticosteroids.

**Table 1:** Demographic characteristics of the study population

Variables	Control group (n = 130)	IPA group (n = 35)	*P* value
Male	95	26	0.87
Age,y	66.4 ± 9.00	65.57 ± 8.90	0.63
History of smoking	113	30	0.85
Steroid treatment	84	32	0.002
Hospitalization history (within 1 month)	14	5	0.56
Admission to ICU	12	4	0.70
*Comorbidity*			
Diabetes mellitus	21	7	0.59
Cardiovascular diseases	17	5	0.85
Extrapulmonary malignant tumor	15	5	0.67
Bronchoscopy	83	28	0.07
*Pulmonary function*			
GOLD 1	23	4	0.71
GOLD 2	34	8	
GOLD 3	51	17	
GOLD 4	22	6	

### Pentraxin 3 levels in plasma and bronchoalveolar lavage fluid

The median [IQR] plasma PTX3 levels in the IPA group (3.74[2.57–5.61]ng/mL) was significantly higher than that in the control group (1.29 [0.62–2.88] ng/mL, *P* < 0.001). Similarly, the median [IQR] level of PTX3 in the BALF of the IPA group was significantly higher than that in the control group (3.88[2.28–8.29]ng/mL vs. 1.58[0.85–2.13]ng/mL, *P* < 0.001).

### Diagnostic accuracy of pentraxin 3 and galactomannan

**Fig. 2:**
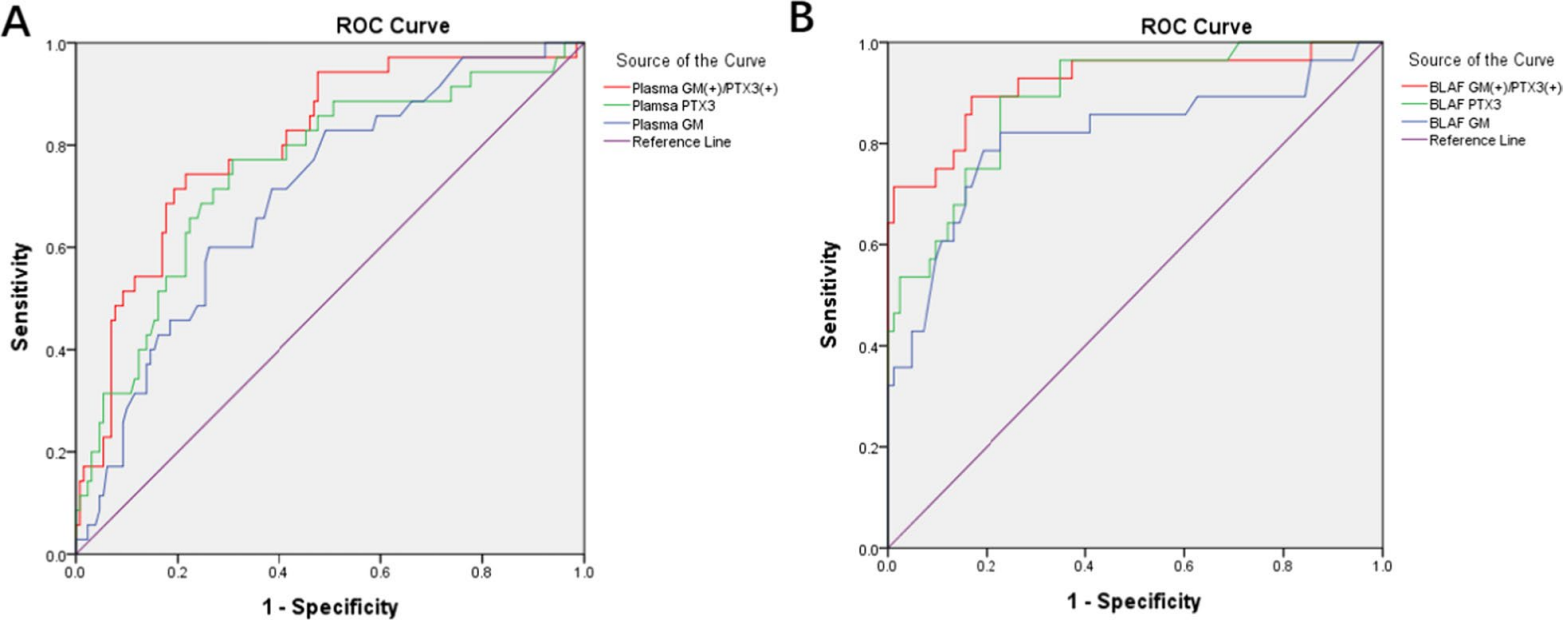
Diagnostic accuracy of pentraxin 3 and galactomannan. **A** ROC curve for plasma GM/PTX3, The area of plasma GM/PTX3/Double(+) under the ROC curve was 0.704/0.751/0.804, respectively. **B** ROC curve for BALF GM/PTX3. The area of BALF GM/PTX3/Double(+) under the ROC curve was 0.813/0.889/0.920, respectively

According to the ROC curve analysis (Fig. 2A), the optimal cutoff value for plasma GM for diagnosing IPA was 0.55, at which the sensitivity and specificity of the test were 60% and 73.8%, respectively (area under the curve [AUC] = 0.704). The optimal cutoff value for plasma PTX3 for diagnosing IPA was 2.57 ng/mL, at which the sensitivity and specificity of the test were 77.1% and 69.2%, respectively (AUC = 0.751). The sensitivity of plasma PTX3 was significantly higher than that of plasma GM (*P* = 0.04), but the difference in the specificity of plasma PTX3 and plasma GM was not statistically significant (*P* = 0.69).

According to the ROC curve analysis (Fig. 2B), the optimal cutoff value for BALF GM for diagnosing IPA was 0.8, at which the sensitivity and specificity of the test were 78.6% and 80.7%, respectively(AUC = 0.813). In optimal cutoff value for BALF PTX3 for diagnosing IPA was 2.16 ng/mL, at which the sensitivity and specificity were 89.3% and 77.1%, respectively. The sensitivity of BALF PTX3 was higher than that of BALF GM, but the difference was no statistical significant (*P* = 0.37).

### Diagnostic accuracy of galactomannan and pentraxin 3 measured in combination

The correlation between PTX3 and GM in patients with IPA is shown in Table 2. In the plasma test, patients who were negative for PTX3 were also negative for GM. Sixty percent of IPA patients were positive for GM and PTX3 in plasma, and 75% of IPA patients were positive for both PTX3 and GM in the BALF samples.

**Table 2:** The correlation between GM and PTX3 in different samples of IPA patients

	GM(+)	GM(−)
Plasma (N = 35)		
PTX3( +)	21	6
PTX3( −)	0	8
BALF (N = 28)		
PTX3( +)	21	4
PTX3( −)	1	2

Double positivity of the plasma GM and PTX3 tests for the diagnosis of IPA in COPD patients had a sensitivity of 60.0% and specificity of 93.8% (AUC = 0.804). In the BALF samples, double positivity of the GM and PTX3 tests in IPA had a sensitivity of 75% and specificity of 94% (AUC = 0.920) (Fig. 2, Table 3).

**Table 3:** The diagnostic values of GM and PTX3 measured in combination

	Plasma GM(+)/PTX3(+) (%)	BALF GM(+)/PTX3(+) (%)
Sensitivity	60	75
Specificity	93.8	94.0
Positive predictive value	72.4	80.8
Negative predictive value	89.7	91.8

## Discussion

PTX3 is a soluble pattern recognition receptor that can be immediately released into the extracellular space in response to inflammation [11]. Several studies have shown that PTX3 plays a key role in innate immunity to *Aspergillus* infections [12, 13]. In this study, the sensitivity of PTX3 was higher than that of GM for the diagnosis of IPA in patients with COPD. Using GM and PTX3 testing in combination is could improve the diagnosis of IPA in patients with COPD.

Previous studies [4] have shown that long-term steroid use is a risk factor for pulmonary aspergillosis. In this study, more patients in the IPA group had a history of corticosteroid use. PTX3 is an acute-phase protein that can be detected within a few hours of inflammation [14]. In this study, the plasma PTX3 levels in the patients with IPA were higher than those of the control group. A previous study found that plasma PTX3 levels were significantly higher in patients with fungal infections, but that C-reactive protein levels did not differ from those of patients without fungal infections [15]. Our previous research is consistent with the above findings [9], In a study of patients without neutropenia Li et al. [16]. The median plasma PTX3 level of aspergillosis patients was 6.97 ng/mL, which is higher than the median plasma PTX3 level of 3.74 ng/mL found in IPA patients in the present study. The reason for the higher median plasma value in the previous study may be that patients with IPA and chronic pulmonary aspergillosis were included, and the patients had more comorbidities, which may have affected the plasma PTX3 level. In Li et al.’s study [16], the ROC curve showed that the optimal plasma PTX3 cutoff value for diagnosing aspergillosis was 2.3 ng/mL, with a sensitivity and specificity of 78.9% and 72.1%, respectively. In the present study, ROC curve analysis found that the optimal plasma PTX3 optimal cutoff value was 2.57 ng/mL, with a sensitivity and specificity of 77.1% and 69.2%, respectively. In this study, the optimal value of serum GM was 0.55, and its sensitivity (60.0%) was significantly lower than that of plasma PTX3, with no significant difference in specificity. Currently, few studies have reported on the diagnostic accuracy of plasma GM testing for diagnosing IPA in patients with COPD. At a cutoff value of 0.5, the reported sensitivity of plasma GM testing has ranged from 11.6 to 90.9%, and the reported specificity has ranged from 66.3 to 100% [17–19]. The plasma GM test sensitivities and specificities recorded in our study were within the ranges reported in previous studies.

In previous studies, BALF was considered a better indicator of lung inflammation than plasma [20]. Currently, the BALF GM test is the best biomarker for the diagnosis of IPA. In this study, according to the ROC curve analysis, the optimal BALF GM cutoff was 0.8, and the sensitivity and specificity were 78.6% and 80.7%, respectively. As the cutoff index increased, the specificity of BALF GM detection increased with the detriment of sensitivity. At a higher GM cutoff value of 1.0, although the specificity was higher (92.8%), the sensitivity was significantly lower (42.9%), which may lead to a delay in diagnosis. Other studies in which ROC curve analysis was applied have reported optimal BALF GM cutoff values ranging from 0.8 to 1.25 in COPD patients with pulmonary aspergillosis [18, 19, 21].One of these studies by Zhang et al. [19], reported the same optimal cutoff value of 0.8 as in the present study, but the sensitivity (88.9%) and specificity (100%) were higher than in this study. The reason for the higher sensitivity and specificity than in the present study is that the patients in Zhang et al.’s study included critically ill patients with COPD, whereas the present study included few critically ill patients. A study in a mixed population of patients with IPA, chronic necrotizing pulmonary aspergillosis, and allergic bronchopulmonary aspergillosis found that the optimal cutoff value was 1.19 [21]. At the optimal cutoff value, the sensitivity (67.9%) was lower than that in our study, and the specificity (89.2%) was higher. The differences in the study populations may have been responsible for the differences in the results. In the study with the highest plasma GM cutoff value of 1.25 [18], most of the patients were ICU patients or received mechanical ventilation. In the study of aspergillosis in patients with non-neutropenia by Li et al. [16], it was also found that the BALF PTX3 was significantly higher in the IPA group than in the control group, similar to our findings. According to the ROC curve in our study, the optimal BALF PTX3 threshold was 2.16 ng/mL, and, the sensitivity and specificity were 89.3% and 77.1%, respectively. In study by Li et al. [16], the ROC curve analysis showed that the BALF PTX3 optimal cutoff was 1.9 ng/mL, with a sensitivity and specificity of 86.3% and 82.5%, respectively. The different results of the two studies are due to the different study populations. Li et al. [16] also compared the sensitivity and specificity of BALF GM and PTX3, and found that the sensitivity of PTX3 was greater than that of BALF GM, and that the specificity was lower than that of BALF GM, as in the present study. However, in our study, the sensitivity of plasma PTX3 and GM was significantly different. These findings suggest that PTX3 is better than GM for the diagnosis of IPA, especially in COPD patients.

In this study, 72.4% of the patients with both plasma GM and PTX3 positivity, and more than 80% of the patients with both BALF GM and PTX3 positivity had IPA. When PTX3 and GM were both positive in plasma or BALF, the specificity for the diagnosis of pulmonary aspergillosis was over 90%. These findings suggest that GM combined with PTX3 can increase the specificity of the diagnosis of IPA in patients with COPD.

## Conclusions

In conclusion, the sensitivity of PTX3 was better than that of GM for the diagnosis of IPA in patients with COPD. Double positivity for GM and PTX3 in plasma or BALF is helpful for the diagnosis of IPA in patients with COPD. The findings have diagnostic implications and need to be validated in a larger cohort.

## Data Availability

The datasets generated and/or analysed during the current study are not publicly available due other manuscripts will be published from this data, but are available from the corresponding author on reasonable request.
